# Novel Approach for Enhanced Scandium and Titanium Leaching Efficiency from Bauxite Residue with Suppressed Silica Gel Formation

**DOI:** 10.1038/s41598-018-24077-9

**Published:** 2018-04-04

**Authors:** Gözde Alkan, Bengi Yagmurlu, Seckin Cakmakoglu, Tobias Hertel, Şerif Kaya, Lars Gronen, Srecko Stopic, Bernd Friedrich

**Affiliations:** 1 IME- Process Metallurgy and Metal Recycling, RWTH Aachen University, Aachen, Germany; 2 MEAB Chemie Technik GmbH, Aachen, Germany; 3 KU Leuven, Department of Materials Engineering, Kasteelpark Arenberg 44, 3001 Leuven, Belgium; 4 IML- Chair of Mineralogy and Economic Geology, RWTH Aachen University, Aachen, Germany

## Abstract

The need of light weight alloys for future transportation industry puts Sc and Ti under a sudden demand. While these metals can bring unique and desired properties to alloys, lack of reliable sources brought forth a supply problem which can be solved by valorization of the secondary resources. Bauxite residue (red mud), with considerable Ti and Sc content, is a promising resource for secure supply of these metals. Due to drawbacks of the direct leaching route from bauxite residue, such as silica gel formation and low selectivity towards these valuable metals, a novel leaching process based on oxidative leaching conditions, aiming more efficient and selective leaching but also considering environmental aspects via lower acid consumption, was investigated in this study. Combination of hydrogen peroxide (H_2_O_2_) and sulfuric acid (H_2_SO_4_) was utilized as the leaching solution, where various acid concentrations, solid-to-liquid ratios, leaching temperatures and times were examined in a comparative manner. Leaching with 2.5 M H_2_O_2_: 2.5 M H_2_SO_4_ mixture at 90 °C for 30 min was observed to be the best leaching conditions with suppressed silica gel formation and the highest reported leaching efficiency with high S/L ratio for Sc and Ti; 68% and 91%; respectively.

## Introduction

European countries started to set deadlines for fueled cars with the new regulations and implementations on CO_2_ emission and fuel consumption of the vehicles for a greener future by Paris Climate Accord (Accord de Paris)^1^, which put light-weighted vehicles and electric cars under spotlight. Titanium (Ti) and scandium (Sc) can be considered as the most potent candidate materials especially for future transportation industry, since they can propose some unique properties for light weight alloys^2–4^. The demand trend for both elements is in a steep increase that’s why these two elements are started to be classified as critical materials. Especially, scandium, which can be used as a tuning metal in aluminum alloys, has limited primary sources and has to be extracted mainly from secondary raw materials or as a by-product of uranium, nickel-laterite or titanium pigment processing. Complex extraction and purification routes as well as the geological scarcity, made the prices of scandium extreme (228 $/g in metallic form)^5^.

Bauxite residue (BR) is the by-product of the Bayer process with 108.7~163.1 million tons of global production as reported in 2015^6,7^. This highly alkaline (pH = 10–12.5) by-product, considered as a valuable secondary resource owing to its promising metal (Fe, Al, Ti, Sc, and REE) content, is stockpiled all over the globe. Hence, valorization of BR has been in the focus of great interest^8–11^. Since the processing route must be tailored in a relation to the geological presence of the BR as a consequence of different mineralogy and association of the phases, several processes have been proposed^12–16^. Hydrometallurgical, pyrometallurgical and the combination of these methods have been previously investigated for the recovery of metals from red mud^16–20^. Since the content of Greek BR comprise valuable amount of scandium (~100 ppm) and significant amount of titanium (~4.3%), it was focused as a candidate secondary raw material for critical metals for future applications.

The recovery of scandium and titanium were mostly examined by direct acid leaching route^21–25^. Reid *et al*. reported the extraction of Sc from red mud via microwave heat treatment followed by 1.5 M H_2_SO_4_ leaching at 90 °C. Application of microwave treatment increased Sc leaching efficiency from 40% to 60%^14^. Another study performed by Borra *et al*. reported a 70% Sc extraction via direct leaching of red mud with 6 M sulfuric acid with low solid-to-liquid ratio of 1/50 at the end of 24 hours^21^. While sulfuric acid was determined as the best candidate for direct acid leaching of both metals, the need of pre-treatment, low solid-to-liquid ratio, high acid concentration and consumption, and low leaching selectivity towards titanium and scandium, remain as the main drawbacks of the proposed leaching routes^21,22,25,26^.

## Current Drawbacks and the Concept for Innovative Leaching Process

**Figure 1:**
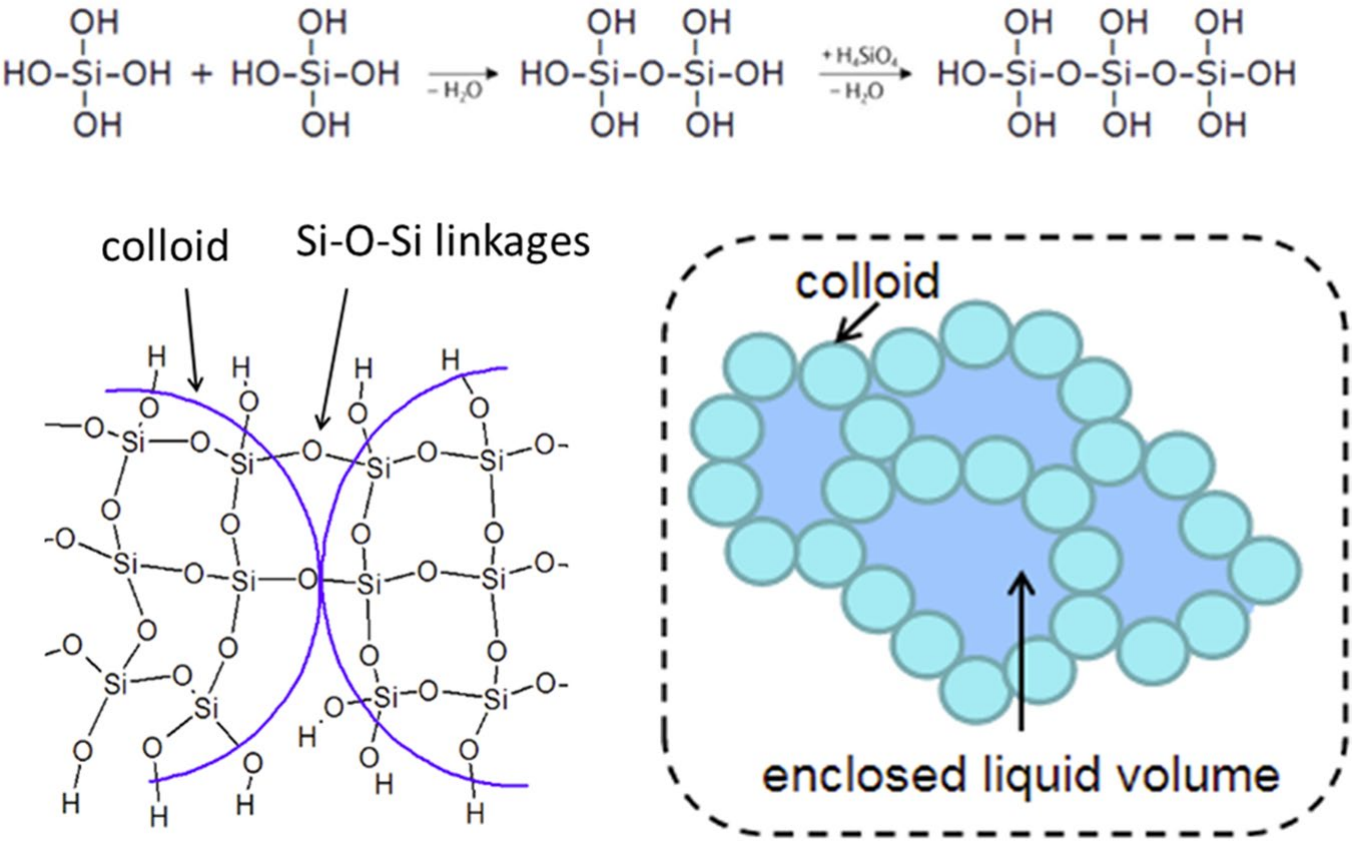
Schematic explanation of silica gel formation in leaching system^28^.

Silica gel formation is one of the most common problems in hydrometallurgical processing of silica containing resources such as BR with 7 wt. % Si content which deteriorates sample handling and solid liquid separation^27^. In acidic conditions (pH < 7), soluble silica is found as silicic acid, Si(OH)_4_, these monomers are connected to each other through Si-O-Si branches and form a polysilicic acid (see Fig. 1), which would afterwards form colloids. When these colloids are connected to each other with entrapped liquid inside, gelation takes place as represented in Fig. 1. Gel formation depends on pH, temperature and ionic strength of the solution.

In previous studies, to tackle the gelation problem, lower solid-to-liquid (e.g. S/L = 1/50), higher acid concentration and leaching temperatures have been proposed^17,21^. However, such acid consumptions decrease the potential of the adaptation into industrial applications. Therefore, in this study, the use of an oxidative leaching condition with higher S/L ratio was examined to change the reaction mechanism of silica compounds by oxidation of dissolved Si ions to prevent their polymerization reaction. Beyond silica gelation problem, such an oxidative atmosphere may also support selectivity of leaching, which is the second drawback of direct acid leaching. Due to high (~40%) iron oxide content of the BR, higher amounts of Fe also dissolve together with the desired elements during acidic leaching conditions and leads to decreased selectivity which in turn results in difficulties during the recovery step of Ti and Sc by precipitation from the pregnant leaching solution (PLS)^29^.

A certain amount of dissolved Fe precipitates with sulfate ions as rhomboclase (H_5_Fe^3+^O_2_ (SO_4_)_2_.2H_2_O) which also traps Sc. Oxidative leaching may suppress iron sulfate precipitation and/or may favor their precipitation into insoluble oxide form. Therefore; it is expected to have a direct impact on Sc leaching efficiency and selectivity via oxidative leaching conditions. Similarly, loss of REE as double sulfate precipitation was reported in the presence of excess sulfate ions. Combination of sulfuric acid with another reagent that would decrease the sulfuric acid consumption may also be beneficial for REE extraction rates.

In brief, in order to achieve a leaching system with less Fe-sulfate precipitation and suppressed silica gel formation, oxidative leaching condition was obtained by use of the H_2_SO_4_:H_2_O_2_ combination, which acts as an oxidizing agent in the presence of H_2_SO_4_^30^. Moreover, H_2_O_2_ and H_2_SO_4_ combination contributes to titanium peroxo sulfate formation which is soluble during leaching and the mechanism of dissolution in PLS can be exemplified via the following chemical reactions^31^;

1 $${{\rm{TiO}}}_{2}+{{\rm{H}}}_{2}{{\rm{SO}}}_{4}- > [{\rm{TiO}}]{{\rm{SO}}}_{4}$$

2 $$[{\rm{TiO}}]{{\rm{SO}}}_{4}+{{\rm{H}}}_{2}{{\rm{O}}}_{2}- > [\mathrm{TiO} \mbox{-} {\rm{O}}]{{\rm{SO}}}_{4}+{{\rm{H}}}_{2}{\rm{O}}$$

Such a particular interaction has not been reported for Al and Fe, which induces the use of peroxide for enhanced selectivity of Ti over Al and Fe during leaching. In order to assure the advantages of H_2_O_2_ addition, varying concentrations of H_2_O_2_ added to H_2_SO_4_ at various S/L ratios and temperatures. Leachates and leach residues were analyzed by ICP- OES, UV-Vis, XRD and QEMSCAN techniques to observe changes in leaching efficiency and mechanism by incorporation of the H_2_O_2_. The most promising leaching parameters were suggested for the efficient and selective Sc and Ti leaching for the valorization of BR. It should be also noted that that H_2_SO_4_: H_2_O_2_ combination has never been utilized for red mud leaching as well as on any other system to prevent silica gelation with increased Ti and Sc recovery.

## Results and Discussion

### Assessment of the H_2_SO_4_ Molarity

Initially, optimum sulfuric acid molarity that will be used in combination with peroxide was determined. Stoichiometric need for complete dissolution of the elements were calculated as approximately 2 M for solid-to-liquid ratio of 1/10. Leaching efficiencies with 1, 2.5 and 4 M of H_2_SO_4_ solutions were investigated in a comparative manner with constant leaching parameters (S/L = 1/10, T = 75 °C, t = 120 min).

**Figure 2:**
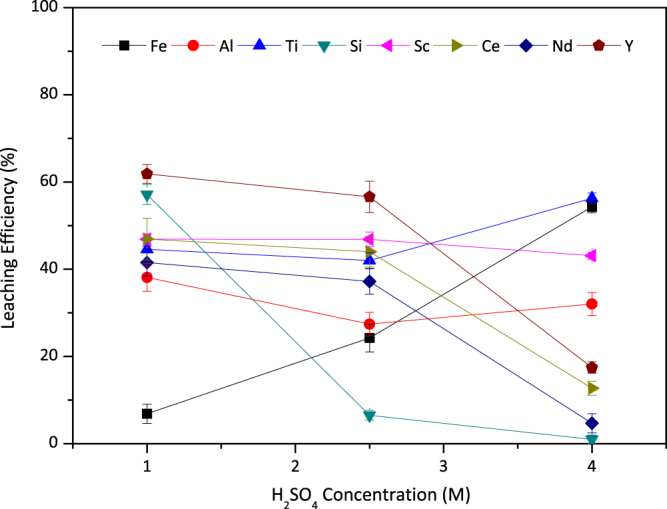
Leaching efficiency of Fe, Al, Ti, Si, Sc and REEs with varying concentrations of H_2_SO_4_ with S/L = 1/10 at 75 °C for 120 min. Error bars represent the standard error of mean for three replicates.

Figure 2 reveals the leaching efficiencies as a function of H_2_SO_4_ molarity for Fe, Al, Ti, Sc, Si and REE. In a general manner, the effect of H_2_SO_4_ molarity was observed to be different for all elements under consideration. With maximum leaching efficiency of 45%, Sc showed almost no dependence on acid molarity. Similarly, there was no significant effect on Ti leaching efficiency. Although the highest leaching efficiency was achieved by 4 M H_2_SO_4_, the decrease of the acid molarity did not yield in a major suppression of Ti recovery. In contrast to Sc and Ti, change of H_2_SO_4_ molarity affected the dissolution behavior Fe, Si and REE dramatically. At low acidity lower amount of Fe oxide dissolution was observed and resulted in very low (less than 10%) Fe leaching efficiency. The dissolution of Fe increased up to 60% by use of 4 M H_2_SO_4_, which is not desired in terms of selective dissolution of Sc and Ti as mentioned previously. On the contrary, the dissolved Si in the PLS was considerably decreased by increasing the acid molarity due to increase in ionic strength as reported previously^32^.

It is also worth to emphasize that, lower H_2_SO_4_ molarity yields in relatively enriched PLS in terms of REE (Y, Nd and Ce). This may be due to favored double sulfate precipitation of REE, in the presence of excess sulfate ions in the system. Despite the independence of Ti and Sc from the acid molarity, selection of the optimum acid concentration is important in terms of Fe, Si and REE elements. Lower acid molarities, especially 1 M H_2_SO_4_, resulted in silica gelation problem, imposing the use of higher acid molarity. On the other hand, suppressed Fe leaching, and enrichment of PLS in terms of REE were favored with lower acid molarities. Beyond that, higher acid consumptions would also decrease industrial application potential, especially due to environmental regulations and economical concerns. When all these issues are considered together, 2.5 M H_2_SO_4_ was selected as the optimal acid concentration and used in subsequent experiments.

### Implementation of the H_2_O_2_

**Figure 3:**
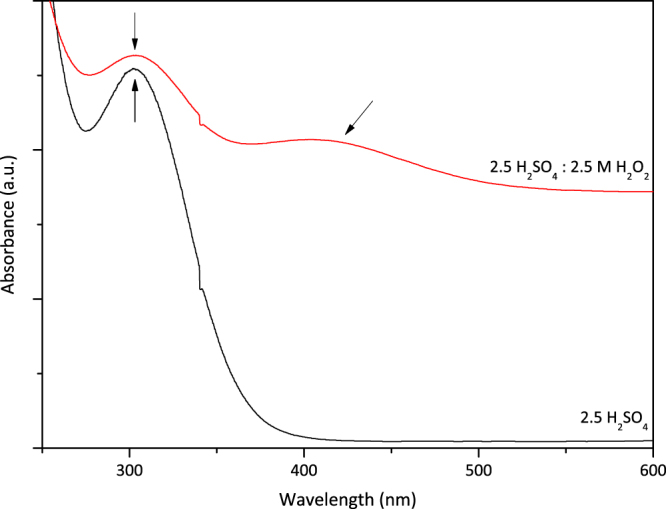
UV- Vis analysis of PLS after H_2_SO_4_ (black curve) and H_2_SO_4_: H_2_O_2_ leaching (red curve).

After determination of the H_2_SO_4_ molarity as 2.5 M, the effect of hydrogen peroxide addition was investigated. At first, to investigate the formation of titanium peroxo sulfate complex, obtained leach solutions, by 2.5 M H_2_SO_4_ leaching and 2.5 M H_2_O_2_:2.5 M H_2_SO_4_ combination, were analyzed and compared by UV-Vis spectroscopy as represented in Fig. 3.

The absorbance at 325 nm observed in both spectra, corresponding to [SO_4_]^2−^, as previously reported^33^. The main difference in the presence of H_2_O_2_ is the absorbance peak at 419 nm, indicated by arrow in Fig. 3, was observed just in the presence of peroxide, which belongs to orange-yellowish colored [TiO-O]SO_4_ complex. Comparative UV-Vis analyses revealed the formation of soluble Ti complex, which may be promising for more efficient and selective Ti leaching.

### Understanding the Effect of H_2_O_2_ Addition on Leaching

**Figure 4:**
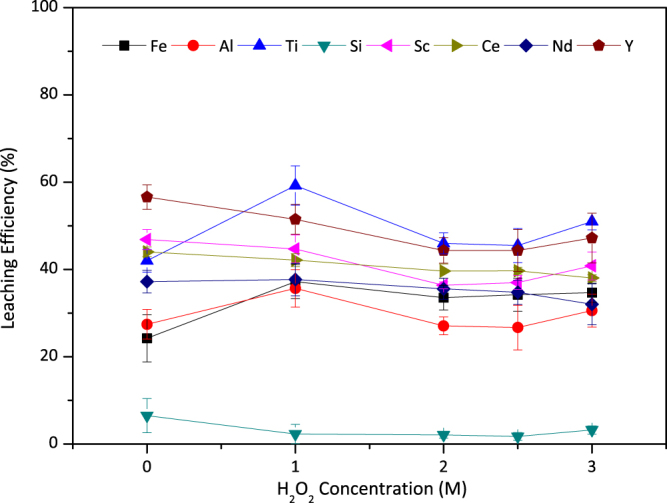
Leaching efficiency of Fe, Al, Ti, Si, Sc and REEs with combination of 2.5 M H_2_SO_4_ and varying molarities of H_2_O_2_ with S/L = 1/10 at 75 °C for 120 min. Error bars represent the standard error of mean for three replicates.

After proving titanium peroxo sulfate formation, a detailed parametric study was performed to understand the effect of hydrogen peroxide addition on leaching mechanisms of the metals. For this purpose, initially, various concentrations of H_2_O_2_ were combined with 2.5 M H_2_SO_4_ with constant S/L = 1/10, at 75 °C for 120 min in a comparative manner. Figure 4 represents the leaching efficiencies of the metals under consideration as a function of H_2_O_2_ molarity.

Figure 4 shows that the use of various concentrations of H_2_O_2_ with 2.5 M H_2_SO_4_ to leach BR at 75 °C for 120 min does not result in any major change in leaching efficiencies of Fe, Ti, Sc and REE. However, it is worth emphasizing that all systems including H_2_O_2_ had almost 0% Si dissolution, while it was around 10% in 2.5 M sulfuric acid leaching. Since during filtration to separate the solid residue and PLS, no gelation problem was experienced as in the case of sulfuric acid, these low values imply precipitation of Si into oxide in the presence of H_2_O_2_.

**Figure 5:**
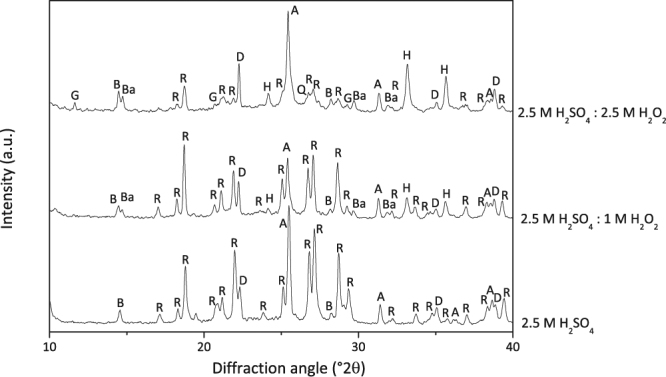
XRD diffractograms of solid residues after leaching with 2.5 M H_2_SO_4_, 2.5 M H_2_SO_4_: 1 M H_2_O_2_ and 2.5 M H_2_SO_4_: 2.5 M H_2_O_2_ (where B: boehmite, R: rhomboclase, D:diaspore, A: anhydrite, Ba: bassanite, H: hematite, G: gypsum, Q: quartz).

In order to understand how H_2_O_2_ modifies leaching mechanisms of metals, solid residues were elucidated in a comparative manner. XRD diffractograms in Fig. 5 represents the phase contents of the solid residues after leaching with 2.5 M H_2_SO_4_, 1 M H_2_O_2_:2.5 M H_2_SO_4_, 2.5 M H_2_O_2_:2.5 H_2_SO_4_.

Although leaching efficiencies do not change significantly, it is possible to observe a gradual change in the phase contents with varying H_2_O_2_ molarity as shown in Fig. 5, which implies the modification of the leaching mechanism. The main difference with H_2_O_2_ incorporation was observed throughout Fe including phases. H_2_SO_4_ leached solid residue includes Fe only in rhomboclase form, which is re-precipitation of Fe ions formed by dissolution of Fe oxide in the BR. However, it was observed that the addition of hydrogen peroxide suppresses the rhomboclase precipitation partially, revealed by the appearance of hematite and decreased intensities of rhomboclase in XRD diffraction patterns. Furthermore, the sample with 2.5 M H_2_O_2_: 2.5 M H_2_SO_4_, quartz phase is detected as can be seen in Fig. 5 which is consistent with ICP-OES analyses revealed almost no Si content in the PLS. It is also worth to note that Ca sulfate phases were also varied with the addition of H_2_O_2_. Sulfate ions that cannot be entrapped by Fe ions, precipitated into various forms of calcium sulfate phases.

These XRD analyses revealed that, the addition of H_2_O_2_ triggered the Fe and Si oxide precipitation, proving the hypothesis mentioned in the motivation chapter. In order to support the XRD findings, with mineralogical associations, same samples were also analyzed by QEMSCAN technique.

**Figure 6:**
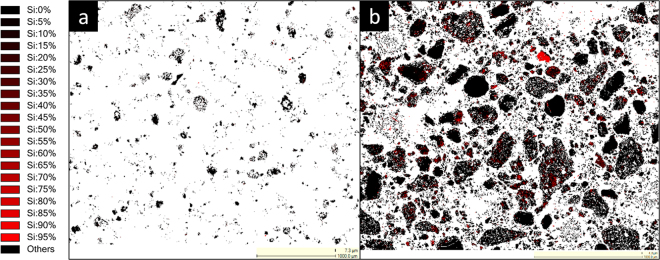
Si elemental mapping of leach residues after leaching with (**a**) 2.5 M H_2_SO_4_ and (**b**) 2.5 M H_2_O_2_: 2.5 M H_2_SO_4_ mixture analyzed by QEMSCAN.

**Figure 7:**
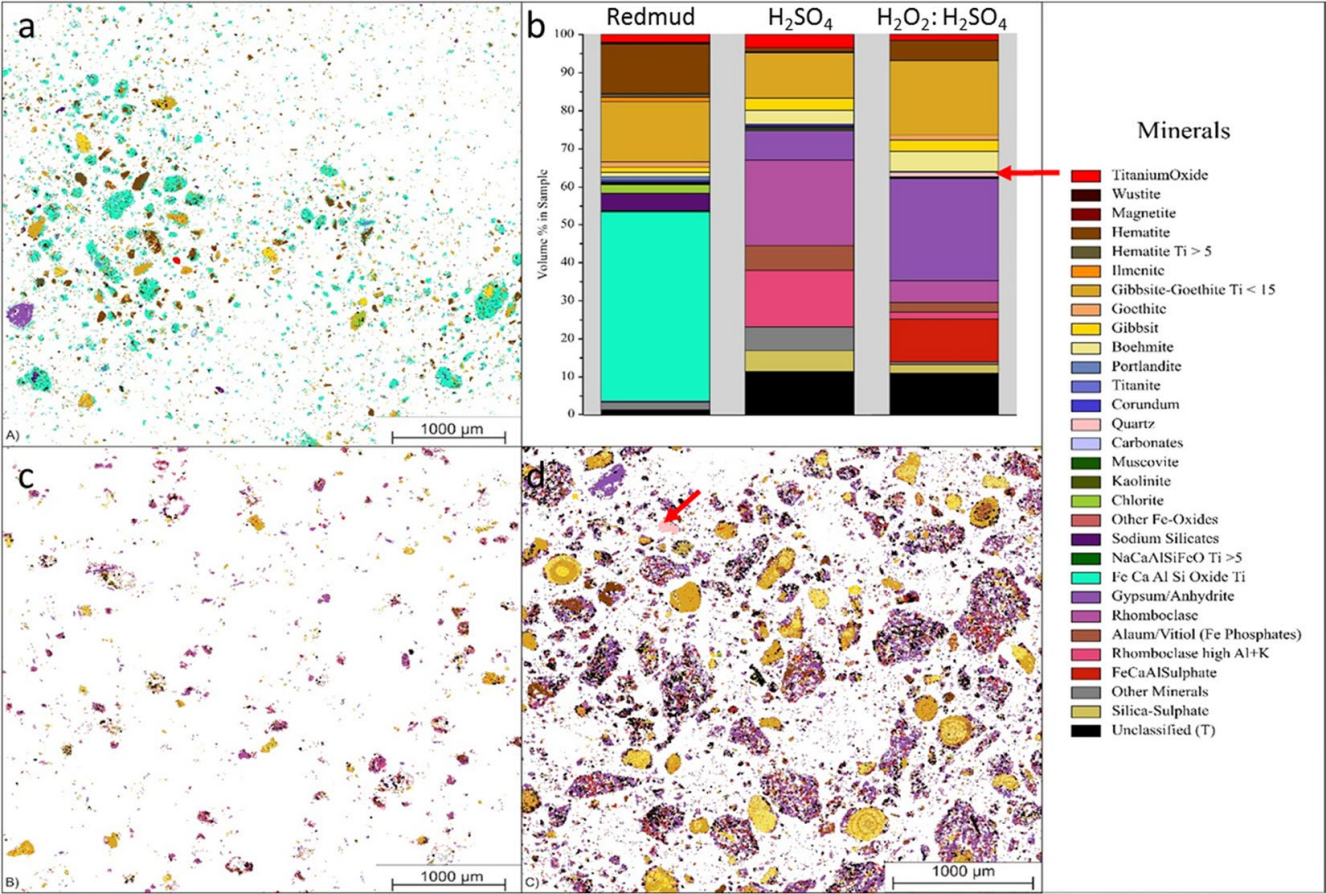
Visual representation of (**a**) the mineralogical distribution of BR, (**b**) phase distribution before and after leaching (**c**) mineralogical distribution of the leach residue after leaching with 2.5 M H_2_SO_4_ and (**d**) mineralogical distribution of the leach residue after leaching with 2.5 M H_2_SO_4_:2.5 M H_2_O_2_ with S/L = 1/10 at 75 °C for 2 h.

Figure 6 represents the elemental mapping of two leached residue samples revealing Si content for direct comparison purposes. Leach residue of 2.5 M H_2_SO_4_ given in Fig. 6a revealed almost no Si, while 2.5 M H_2_O_2_:2.5 M H_2_SO_4_ residue contained significant amount of Si. In parallel with ICP-OES and XRD findings; QEMSCAN study on leach residues also underlined the enrichment of residue in terms of Si. In order to reveal in which phase Si precipitates and results in the leach residue, phase mapping of BR, residues after leaching with 2.5 M H_2_SO_4_ and 2.5 M H_2_O_2_:2.5 M H_2_SO_4_ are represented in Fig. 7.

The scans shown in Fig. 7, where each color corresponds to a different mineral, revealed very distinct distribution within three samples. BR which is represented in Fig. 7a, exhibited Fe, Ca, Al and Si oxide, which is a highly non stoichiometric intergrowth oxide as reported previously^34^. After leaching with H_2_SO_4_, there was not any Si mineral detected. However, when peroxide is incorporated, as indicated by arrows on Fig. 7b and d, precipitation of quartz is favored, as consistent with XRD analyses. Another critical difference between two different residues was observed in terms of Fe containing minerals. Almost all Fe, in the leach residue of H_2_SO_4_ leaching was detected to be in the rhomboclase phase in parallel with the XRD results. However, the incorporation of H_2_O_2_ resulted in a great suppression of the rhomboclase phase and increased the content of iron oxide phase as can be observed from Fig. 7b which is also in agreement with the findings of XRD analyses.

XRD, QEMSCAN and ICP-OES analyses exhibited a great consistency in terms of the effect of hydrogen peroxide addition on leaching mechanism. The use of H_2_O_2_:H_2_SO_4_ in combination induced the precipitation of Fe and Si oxides and therefore seems promising in terms of selectivity and suppressed gelation problem. Although the results seems better in leaching efficiency point of view with the implementation of 1 M H_2_O_2_ with H_2_SO_4_, a significant decrease in rhomboclase phase as well as the quartz formation was observed in both XRD and QEMSCAN analysis when 2.5 M H_2_O_2_ is used. With the formation of hematite instead of rhomboclase, Sc co-precipitation will be limited since rhomboclase is one of the major phases entrapping scandium during re-precipitation. Additionally, the formation of quartz, which can be seen from XRD and QEMSCAN, is the needed phase to prevent Si-gel formation during and/or after leaching process. Hence, to investigate the dependence of that system on time, temperature and solid to liquid ratio, a controlled parametric study was conducted by the use of 2.5 M H_2_O_2_:2.5 M H_2_SO_4_.

### Assessment of the Leaching Duration

**Figure 8:**
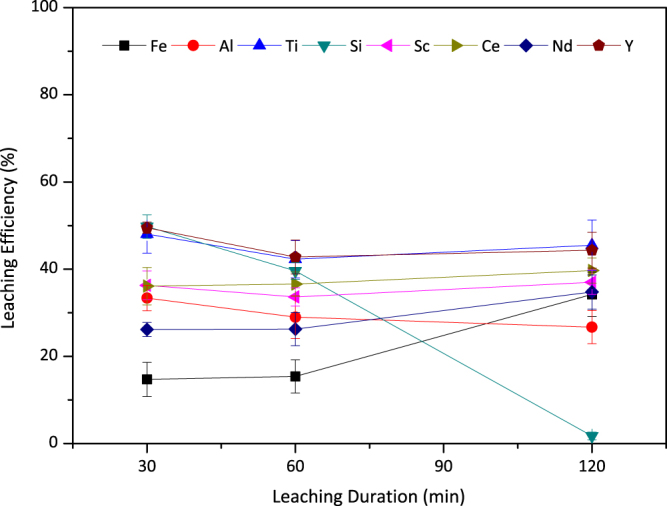
The effect of leaching duration on leaching efficiencies of Fe, Al, Ti, Si, Sc and REEs when 2.5 M H_2_SO_4_:2.5 M H_2_O_2_ mixture is used with S/L = 1/10 at 75 °C. Error bars represent the standard error of mean for three replicates.

As reported by Habashi, leaching process is generally controlled by diffusion and therefore it is expected to yield in higher efficiencies with increasing durations^35^. To reveal the effect of leaching duration on leaching efficiency of Sc, Ti, REEs as well as the major impurities such as Fe, Al or Si, various leaching durations were monitored. Figure 8 shows that, the duration of the process showed minor effect on leaching of the elements present in the BR. The rate controlling step was previously suggested as diffusion of the leaching agent through boundary layer, with which this stable behavior showed a consistency^14^. The concentration of Si was observed to be decreasing noticeably with the time since the oxidation reaction of the Si took place due to the decomposition of H_2_O_2_. Formation of this oxidation product, quartz, will be finalized spontaneously even if the leaching process is stopped at 30 minutes, since there will still be free oxygen to oxidize Si in the solution. Consequently, the optimum duration of the leaching was assessed as 30 minutes for leaching at 75 °C.

### Assessment of the Solid to Liquid Ratio (S/L)

**Figure 9:**
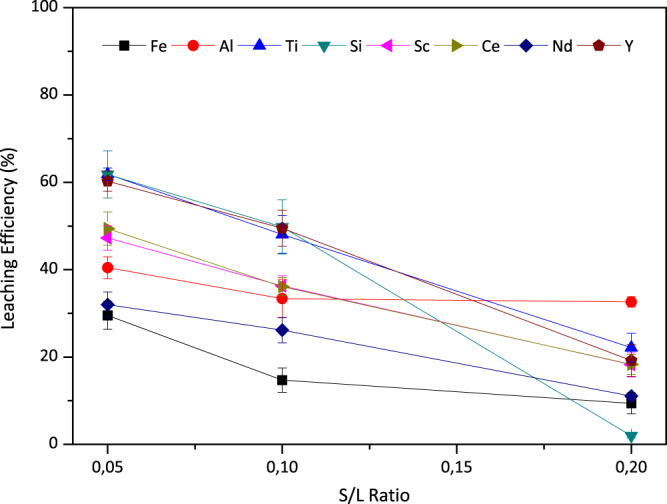
The effect of solid-to-liquid ratio (S/L) on leaching efficiencies of Fe, Al, Ti, Si, Sc and REEs when 2.5 M H_2_SO_4_:2.5 M H_2_O_2_ mixture is used at 75 °C for 30 minutes. Error bars represent the standard error of mean for three replicates.

Figure 9 presents the effect of solid to liquid ratio on leaching efficiency of the constituent elements present in the BR. As it can be seen from the figure, as S/L ratio is decreased, leaching efficiencies increase. The caustic nature of the BR increases the acid need during leaching; hence, it was expected to have higher leaching efficiencies with increasing amounts of the acid introduced. Although, 1/20 ratio seemed to be the best in terms of efficiencies, the acid consumption during the process was increased a lot while the concentration of the Sc and REEs decreased correspondingly, which will complicate the recovery processes after leaching. Therefore, the optimal S/L ratio for this leaching was determined to be 1/10 despite the decrease of Sc leaching efficiency from 47% to 37% and Ti from 62% to 48%.

### Assessment of the Leaching Temperature

**Figure 10:**
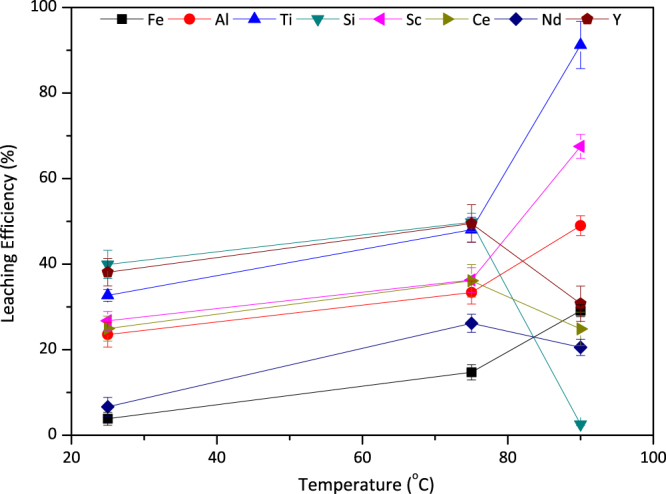
The effect of leaching temperature on leaching efficiencies of Fe, Al, Ti, Si, Sc and REEs when 2.5 M H_2_SO_4_:2.5 M H_2_O_2_ used with S/L = 1/10 for 30 minutes. Error bars represent the standard error of mean for three replicates.

It was reported that an increase in the temperature, increases the rate of dissolution, therefore has an important effect on leaching efficiencies^35^. The effect of temperature on leaching can be seen from the Fig. 10. When the leaching was performed at room temperature, insufficient and low leaching efficiencies were noted.

With the increase in leaching temperature up to 75 °C, all metals exhibited, in a consistency, a slight increase in leaching efficiencies. However, further increase to 90 °C yielded in significant increase for other metals while a great suppression in Si leaching efficiency. This can be explained by quartz formation mechanism in the presence of H_2_O_2_. Higher temperatures favor the decomposition rate of H_2_O_2_ and therefore even at early reaction time (30 min), dissolved Si in leachates is precipitates into quartz and precipitated to the leach residue as revealed in QEMSCAN analyses in Fig. 6.

The elements under the main focus, Sc and Ti, showed major improvements when the temperature was increased to 90 °C. Leaching efficiencies were 32% and 27% at room temperature, 48% and 37% at 75 °C and 91% and 68% at 90 °C for Ti and Sc respectively. To our best knowledge, these leaching efficiencies are one of the best among the previously reported for Ti and Sc in mineral acid leaching with having high S/L ratio, short leaching duration and without any pre-treatment. This drastic increase stems from the rapid decomposition of H_2_O_2_ and more aggressive leaching environment at 90 °C. The rate of formation of the titanium peroxo sulfate complex increases, thus the Ti leaching efficiency peaked at high leaching temperature. Since Sc mainly hosted by hematite and goethite minerals and minor in Ti minerals in BR, increase in the dissolution of those phases directly has a positive impact on the dissolution of Sc^36^. To further increase the Sc efficiency, all of these Sc hosting minerals have to be completely dissolved in the PLS. Another possible reason for the rise in Sc amount in the liquor is the dissolution of hematite with sulfuric acid and release of the Sc hosted by the mineral at the beginning of the process and re-precipitation of the Fe in the PLS as hematite instead of rhomboclase due to excess oxygen in the media in the later stages. So, Fe in the solution was limited while most of the Sc inside the Fe-related minerals was relocated into the acidic solution. Unlike Sc, the leaching efficiencies of the REEs were observed to be decreased with increasing temperature because of the inverse solubility of REE sulfates.

### Comparison with Other Oxidative and Reductive Leaching Ambient

**Figure 11:**
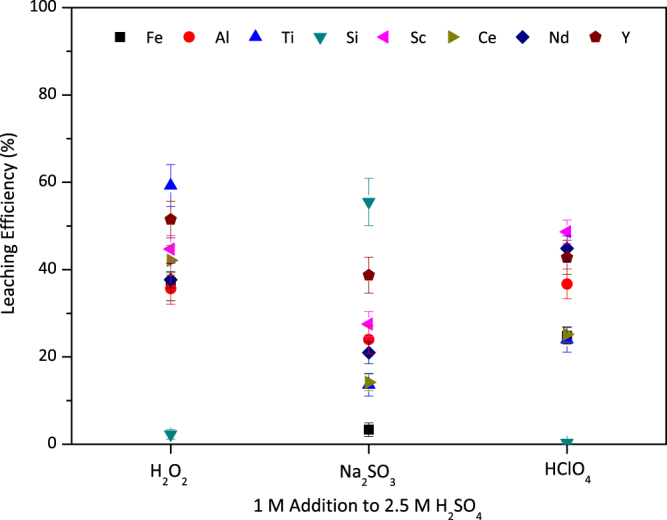
Comparison of 1 M addition of oxidative (H_2_O_2_ and HClO_4_) and reductive (Na_2_SO_3_) into 2.5 M H_2_SO_4_ at 75 °C for 120 min with a S/L = 1/10. Error bars represent the standard error of mean for three replicates.

The effect of oxidative and reductive environment during leaching was investigated and summarized in Fig. 11. It can be seen that while the oxidative media has a coherency in terms of leaching efficiencies, there are distinct differences between oxidative and reductive media. Oxidation and precipitation of Si by the addition of H_2_O_2_, was also confirmed by the addition of another oxidizing agent, HClO_4_. On the contrary, highest leaching efficiency of Si was observed under reductive leaching conditions. When the Si containing mineral in BR is considered, which is highly non-stoichiometric and oxygen deficient, as reported previously, it is expected to form a highly stable quartz phase when oxygen is introduced to the system. Since, Al, Fe and Ti dissolutions were observed to be low when Na_2_SO_3_ was introduced to the system; it was expected to have lower Sc yields in this media than the oxidative ones. The major difference between H_2_O_2_ and HClO_4_ additions is the concentration of Ti in the solution, which is another confirmation of the formed Ti-peroxide interaction.

### Proposed Reaction Mechanism

**Figure 12:**
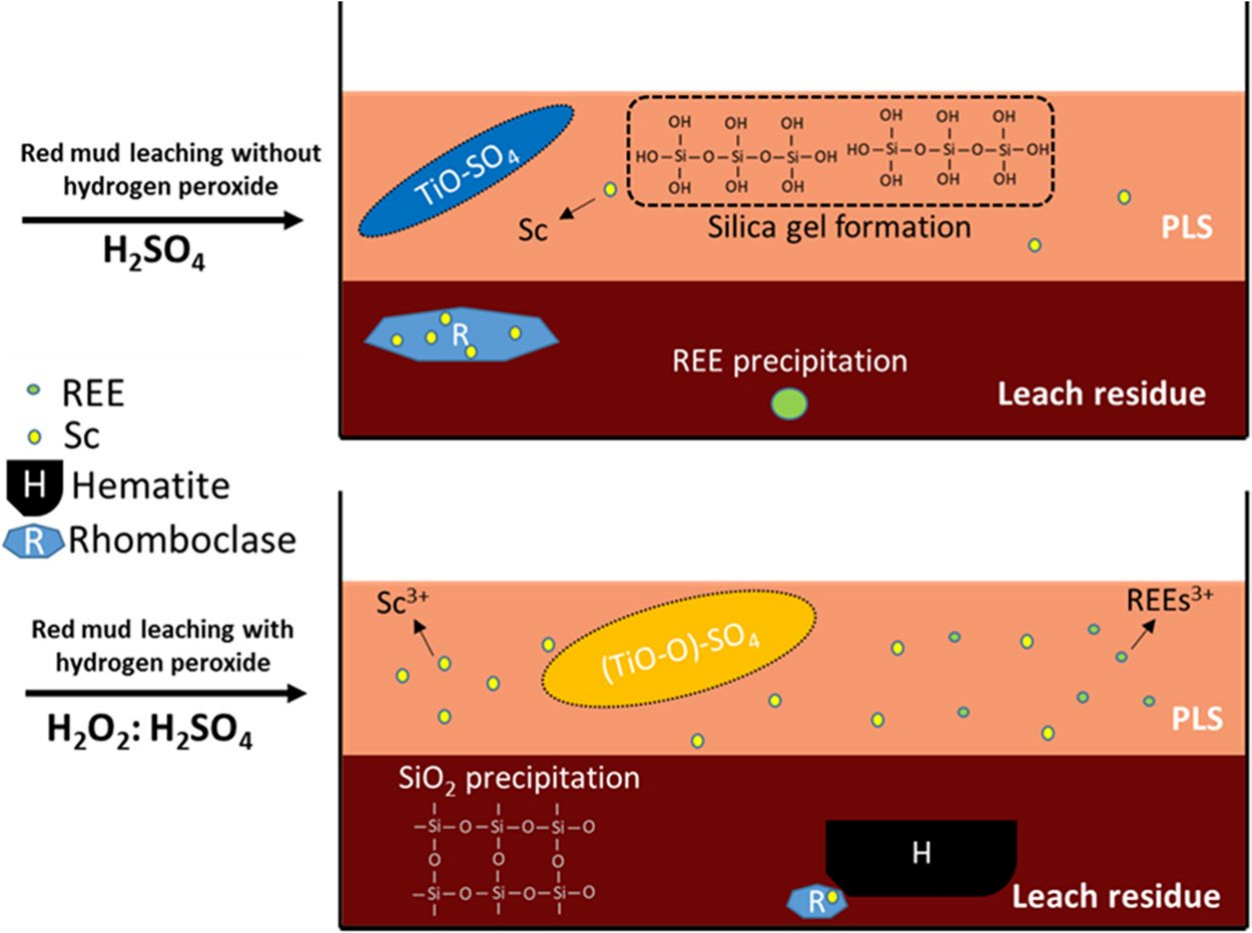
Overview and comparison of the H_2_SO_4_ and H_2_SO_4_: H_2_O_2_ leaching mechanisms.

The mechanisms and the major differences between the use of H_2_SO_4_ alone and H_2_SO_4_ with H_2_O_2_ can be seen from Fig. 12.

Previously, the use of sulfuric acid for direct leaching of the BR was proven to be the best among other mineral acids. Nevertheless, various disadvantages still exist to be solved. For instance, silica gel formation (the need for high acid concentration or low S/L ratios to cope with silica gel formation), low selectivity towards Ti, Sc and REEs and the co-precipitation of Sc in rhomboclase phase can be counted as the significant drawbacks of the direct H_2_SO_4_ leaching of the BR.

Implementation of hydrogen peroxide, not only provides an oxidative atmosphere which forces the dissolved Si to precipitate as quartz phase and prevents the formation of Si-gel, but also forms titanium peroxo sulfate complex yielding in a high Ti leaching efficiency. Furthermore, this oxidative leaching media, decreases the amount of rhomboclase phase which entraps Sc during precipitation and favors the formation of hematite during leaching. Consequently, leaching efficiencies of Ti, Sc and REEs can be enhanced by the combinative use of hydrogen peroxide during leaching.

## Conclusions

The operational parameters were investigated and the addition of 2.5 M H_2_O_2_ into 2.5 M H_2_SO_4_ was decided to be the best leaching condition to have favored quartz formation with a suppressed rhomboclase precipitation. Since the leaching reactions mainly controlled by diffusion, no significant increase in the efficiencies were observed after 30 minutes of leaching. To have high S/L ratio and to decrease the consumption of acid, different S/L ratios were compared. Even though low S/L ratios granted higher efficiencies due to caustic nature of BR, 1/10 ratio found to be the optimal condition. 90 °C was found to be the best leaching temperature for faster Ti complex formation, faster kinetical observation of SiO_2_ precipitation as well as more aggressive environment resulting by faster decomposition of H_2_O_2_. When these optimal operational parameters were implemented, one of the highest reported leaching efficiencies for both Ti and Sc can be achieved at high S/L ratio. The motivations behind this study were also verified with a comparative experimentation of oxidative (H_2_O_2_ and HClO_4_) and reductive (Na_2_SO_3_) additions to the leaching media. While Si gel was not formed in oxidative environment, high Ti efficiency was only achieved when H_2_O_2_ was introduced into the system.

## Methods

The BR used in this study was obtained from Aluminum of Greece S.A. The composition of the bauxite residue is determined by subjecting it to lithium borate fusion, and analyzed using ICP-MS/AAS which can be found in Table 1.

**Table 1:** Chemical composition of the BR sample.

Major Compounds	wt.%	Minor Compounds	ppm
Fe	29.6	La	110
Al	8.6	Ce	380
Ca	8.3	Sc	120
Si	3.3	Nd	100
Ti	2.6	Y	80
Na	2.8		
Others	32.1		
LOI	12.7		

Leaching experiments were carried out in a glass reactor and heating-stirring were controlled by use of a hot plate with magnetic stirrer. BR was washed with de-ionized (DI) water and dried at 90 °C and hand milled to ease leaching. After that, it was fed into the reactor, containing preheated sulfuric acid or its combination with hydrogen peroxide. Mixture of sulfuric acid with hydrogen peroxide should be carefully prepared by slowly adding hydrogen peroxide into sulfuric acid to control the rapid exothermic reaction. The effect of the acid concentration and hydrogen peroxide addition on leaching efficiency was investigated at a set temperature of 75 °C, 250 rpm stirring speed, and S/L ratio of 1/10. Leaching experiments were performed under atmospheric pressure for 30, 60, 90 and 120 minutes. Leaching experiments were repeated three times independently to ensure the accuracy of the calculations and the analysis. Errors for each sample were calculated, which stayed in the range from 0.3% to 6.2% for all samples. Samples from leach liquor were vacuum filtered and leach residues were dried overnight at 90 °C. Leach residues were investigated by XRD, SEM and QEMSCAN techniques to reveal the phase, morphology, and microstructure. In order to evaluate leaching efficiency, leachates were analyzed by ICP-OES technique. For QEMSCAN® analysis, the acceleration voltage and sample current were set to 25 kV and 10 nA at a working distance of 13 mm, respectively. The point spacing was set to 7.5 µm and 2000X -ray counts were recorded per each step. Phase interpretation and further image analysis, like phase mapping, modal composition and elemental mapping, were performed by use of the iDiscover software suite (FEI).

XRD analyses were performed by use of the Bruker D8 Advance diffractometer, which uses Bragg- Brentano geometry and θ-θ synchronization for X-ray tube and the detector. 10°–80° (2 θ) were scanned with a 5°/min scanning rate. The generator voltage was 40 kV and current was 40 mA.

The datasets generated during and/or analyzed during the current study are available from the corresponding author on reasonable request.
